# Proceedings of the 6th Annual United States Army Institute of Surgical Research Summer Undergraduate Research Internship Program 2018

**DOI:** 10.1186/s12967-018-1671-8

**Published:** 2018-11-19

## I1 Proceedings of the United States Army Institute of Surgical Research Summer Internship Program 2018

### Austin Bareis^1^, Xiaowu Wu^2^, Daniel N. Darlington^2^, Andrew P. Cap^2^, Christopher R. Bologna^3^, Johan A. van Nispen^4^, Kim E. Hildreth^2^, David Barraza^2^, Charnae E. Williams^2^, Michael A. Dubick^2^, Ivo P. Torres Filho^2^, Brittny L. Garcia^5^, Megan E. Manos^5^, Misty M. Strain^2^, Roger Chavez^2^, David Trapolsi^2^, Alex Trevino^2^, Thomas Garza^2^, Stephen Crimmins^2^, Charlotte J. Winkler^2,6^, Belinda I. Gómez^2^, Matthew K. McIntyre^2^, Tony Chao^2^, Joshua S. Little^2^,, David M. Burmeister^2^, Grace Chu-Yuan Chu^2,7^, Tiffany C. Heard^2^, Joshua A. Fahy^1, 2^, Deana A. Apple^2^, Katy Cohen^2^, Jeremy Pamplin^2^, Maria Serio-Melvin^2^, Sena R. Veazey^2^, Kerry E. Gonyeau^8^, Emily A. Howard^9^, Thomas H. Edwards^2^, Laura L. F. Scott^2^, Jacque Parker^10^, Kelly Hall^11^, Laurynn Garcia^12^, Christopher Delavan^2^, Carolina Cantu^2^, James A. Bynum^2^, Maryanne C. Herzig^2^, Barbara Christy^2^, Luke A. del Balzo^13^, Maria E. Ramos^13^, Bunyen Teng^2^, Jeffrey D. Keesee^2^, Josue Garciamarcano^2^, Natasha M. Sosanya^2^, Sirima Tongkhuya^2^, Lucy J. Shaffer^14^, Christine J. Kowalczewski^2^, Robert Christy^2^

#### ^1^University of Arkansas, Fayetteville, AR 72701, USA; ^2^US Army Institute of Surgical Research, JBSA Fort Sam Houston, TX 78234, USA; ^3^Louisiana State University, Baton Rouge, LA 70803, USA; ^4^Washington University in Saint Louis, St. Louis, MO 63130, USA; ^5^ University of Texas at Austin, Austin, TX 78705, USA; ^6^Yale University, New Haven, CT 06520, USA; ^7^University of Michigan, Ann Arbor, MI 48109, USA; ^8^Texas Tech University, Lubbock, TX 79409, USA; ^9^Texas A&M University College of Veterinary Medicine, College Station, TX 77843, USA; ^10^Holland Memorial Military Working Dog Hospital, JBSA-Lackland AFB, TX 78236, USA; ^11^University of Minnesota College of Veterinary Medicine, St. Paul, MN 55108, USA; ^12^Duke University, Durham, NC 27708, USA; ^13^Emory University, Atlanta, GA 30322, USA; ^14^Oregon State University, Corvallis, OR 97333, USA

##### **Editors: **Lauren Cornell^2^ and Whitney Greene^2^

###### **Correspondence: **Lauren Cornell (lauren.e.cornell.ctr@mail.mil)

*Journal of Translational Medicine *2018, **16(Suppl 3):**I1

**Program details:** The United States Army Institute of Surgical Research (USAISR) was established in 1943, with a mission to develop state-of-the-art technologies to optimize the quality of combat casualty care. Since then, the USAISR has been involved across the world to improve burn techniques as well as give humanitarian aid. With one of the world’s top burn flight crews, the USAISR is a top innovator in its field recognized for major technological advancements. Recently developed programs have allowed United States based students to work with the researchers involved in these past and recent advancements. In 2012, the USAISR initiated the Summer Undergraduate Internship Program to expose high achieving undergraduate students to real life applications of their respective fields of study. Students work with Department of Defense (DoD) research scientists on cutting-edge biomedical research to address complex medical challenges faced by Service Members injured in combat.

Fourteen students were selected out of hundreds of applicants to participate in the 2018 summer program. For 10 weeks, from June 4 to August 10, 2018, students were paired with research mentors in the following task areas: hemorrhage control and resuscitation, blood and coagulation, burn injury, comprehensive trauma care, intensive care, pain, multi organ support technology, and veterinary support. The interns participated in a variety of activities, including: aiding in research, shadowing physicians during rounds in the USAISR Burn Center Intensive Care Unit, touring the Center for the Intrepid, gardening at the Warrior and Family Support Center, touring the hyperbaric medicine chamber, participating in a weekly research journal club, and attending various other activities related to the field of medical research. Students learned to design experiments, analyze results, and present data. During their time in the program, the interns learned more about potential careers in research, medicine, and military service. Following completion of the 10 week internship program, students presented their completed research projects in the form of a poster session.

**Eligibility:** Applicants to the USAISR Internship Program must have finished their first year of undergraduate studies in an accredited bachelor’s degree program. Applicants that pursue Science, Technology, Engineering, and Mathematics (STEM) majors are preferred. All applicants must be current US citizens to be accepted into the program.

**Meeting format:** At the end of the 10-week program, the interns presented the results of their research to the USAISR, Naval Medical Research Unit-San Antonio, and the San Antonio Military Medical Center scientific community. Attendees included military personnel, clinicians, research fellows, and principal investigators (PIs).

**Awards:** Upon successful completion of the program, interns were awarded with Certificates of Appreciation by Dr. David Burmeister.

**Fig. 1:**
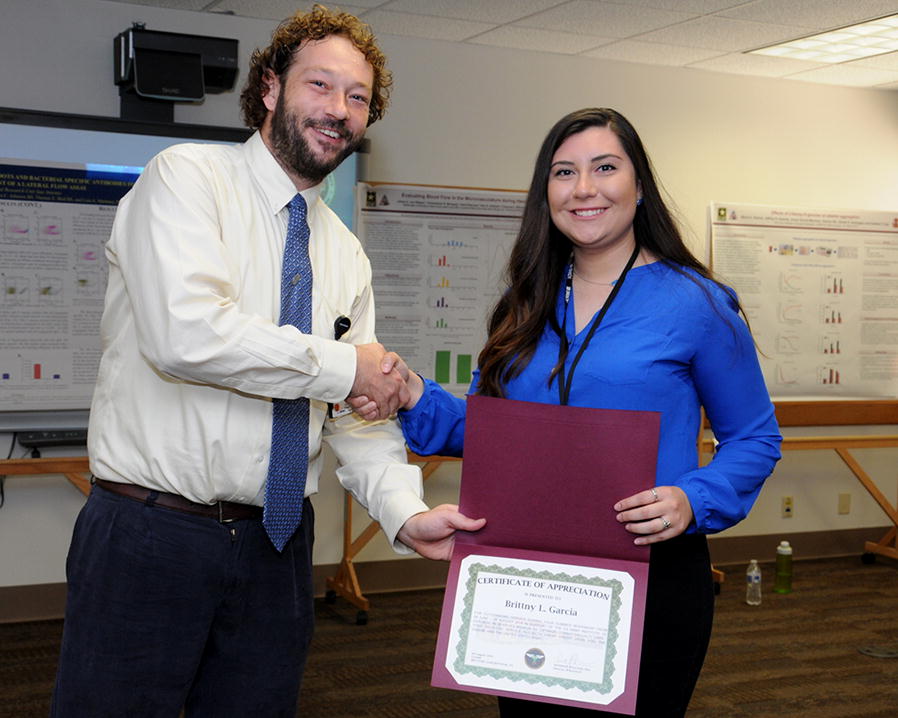
Dr. David Burmeister (left) presents award to student after completion of internship and poster session (Photo Credit: SGT Zeyar Htut)

**Fig. 2:**
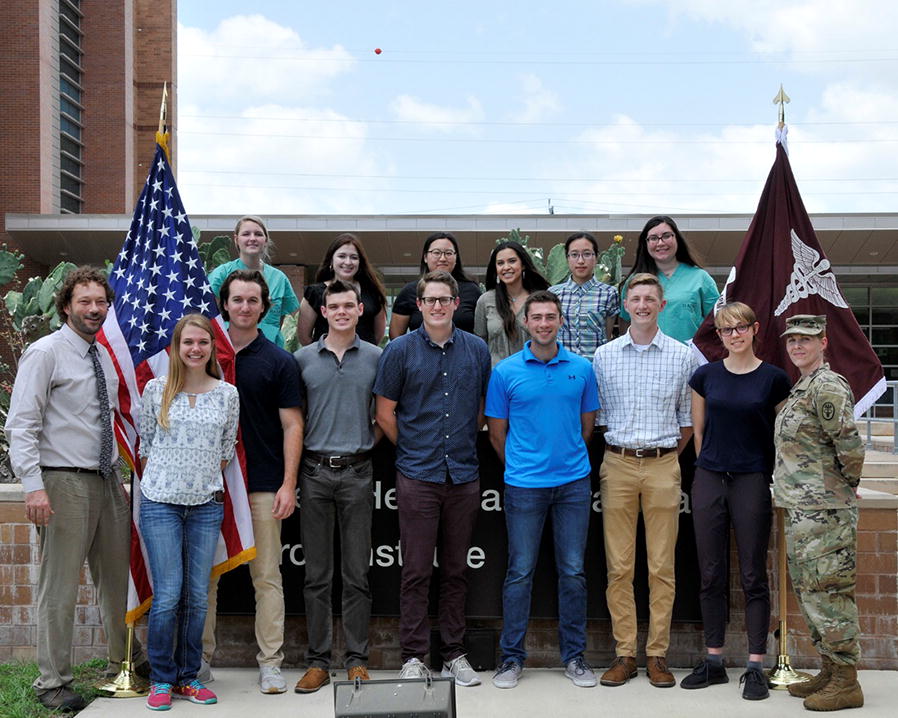
Summer interns outside the USAISR with Dr. David Burmeister (lower left corner) and Captain Melissa Kottke (lower right corner) (Photo credit: U.S. Army Photo by Dr. Steven Galvan, US Army Institute of Surgical Research Public Affairs)

**Conclusions:** The USAISR Summer Internship Program provides an opportunity for students to experience research conducted at a military research facility. The students receive mentorship and guidance from scientific investigators who understand the complexity of combat injuries sustained by military service members. Students acquired thorough understanding of safety protocols and laboratory skills specific to their respective field of study. Students developed skills related to composing and presenting scientific research as well as understanding the underlying basis of scientific articles. These skills were improved with assistance and oversight from the USAISR research staff. Students experienced genuine interprofessional cooperation between scientific research professionals amongst their various research projects, which is crucial to preparing them for their future professions in medicine and biomedical research. Students learn safety associated with the handling of hazardous chemicals. Students also learn how to perform specific laboratory techniques such as western blot analysis, histology and immunocytochemistry, and proper use of microscopes. Skills learned in the following areas such as hemorrhage control and resuscitation, blood and coagulation, burn injury, comprehensive trauma care, intensive care, pain, multi organ support technology, and veterinary support help students relate what they have learned in their studies to real life experiences. Since 2016, the students have written and submitted their abstracts for publication in this journal. This USAISR Summer Internship program grows with each new academic year, allowing students more comprehensive exposure to military biomedical research. In total, this program provides the students with real world scientific research experience, so they may use to develop their educational and career goals.

**Acknowledgements:** We express our gratitude to the USAISR Research Directorate for their sponsorship of the internship program and this publication. We would like to thank Director of Research Dr. Anthony Pusateri, Deputy Director of Research Captain Melissa Kottke, Ms. Melinda Scott, summer intern program coordinator Dr. David Burmeister, intern staff mentor Dr. Amit Aurora, and all the PIs, as well as the other talented research staff members who committed their time to mentoring students. We would also like to recognize Dr. Steven Galvan and SGT Zeyar Htut for their work in capturing the images for this publication. The research done by the summer interns at USAISR was supported in part by an appointment to the Student Research Participation Program by the Oak Ridge Institute for Science and Education through an interagency agreement between the U.S. Department of Energy and United States Army Medical Research and Material Command (MRMC). We thank our institutional editors, Lauren Cornell, M.S. and Whitney Greene, Ph.D. for founding and editing this student publication program.

**Disclaimers:** Opinions contained within are the private views of the authors, not to be interpreted as official or reflecting the views of the Department of Defense as well as Departments of the Army. The following research below is funded by the US Army Medical Research and Material Command. The following studies have been conducted under military protocols, reviewed, and approved by the US Army Medical Research and Material Command Institutional Review Board in accordance with the necessary protocols. Research was conducted in compliance with the Animal Welfare Act, the implementing Animal Welfare Regulations, and the principles of the Guide for the Care and Use of Laboratory Animals, National Research Council. The facility’s Institutional Animal Care and Use Committee approved all research conducted in this study. The facility where this research was conducted is fully accredited by AAALAC International.

**Funding:** Publication of this article was funded by the USAISR.

**Consent:** The authors have written informed consent from all members in the provided images.

**Application website:** Link to the application website can be found here https://www.orau.org/maryland/

## P1 The impact of bradykinin receptor antagonist on tissue edema in trauma/hemorrhagic shock-a pilot study

### Austin Bareis^1^, Xiaowu Wu^2^, Daniel N. Darlington^2^, Andrew P. Cap^2^

#### ^1^University of Arkansas, Fayetteville, AR 72701, USA; ^2^US Army Institute of Surgical Research, JBSA Fort Sam Houston, TX 78234, USA

##### **Correspondence: **Andrew P. Cap (andrew.p.cap.mil@mail.mil)

*Journal of Translational Medicine *2018, **16(Suppl 3):**P1

**Introduction:** Acute traumatic coagulopathy (ATC) has a fibrinolytic component, characterized by an elevation in plasma d-dimers [1] and tissue plasminogen activator (tPA) [2]. Severe trauma in rats also causes elevation in tPA, plasmin activity and d-dimers [3] with a subsequent fall in clot strength [4]. Plasmin is known to generate bradykinin, by activating factor XII and kallikrin. Bradykinin is known to be involved in edema in tissues, including the brain [5, 6]. Bradykinin receptor blockade may have therapeutic benefits in reducing tissue edema after trauma, hemorrhage and resuscitation.

**Objective:** Determine if blockade of bradykinin receptors attenuates the tissue edema in a rat model of trauma/hemorrhagic shock.

**Methods:** Sprague–Dawley rats (350–450 g) were anesthetized with isoflurane. Polytrauma was induced by laparotomy, and gentle crush of intestines, right and medial liver lobes, skeletal muscle (right hindlimb), femur fracture and 40% hemorrhage. At 45 min, the rats were given bradykinin receptor antagonist (200 μl, HOE140, 0.5 mg/kg) (n = 4/each group), followed by resuscitation with Lactate Ringer’s solution (20% of blood volume) at 1 h. Two normal rats without any surgical procedure were treated as normal control. At 2 h after trauma (1 h after resuscitation), the rats were euthanized, and tissues were harvested. The tissues were weighed wet and dry (37 °C oven for 10–14 days).

**Results:** We found that the water content (ml/g tissue) is different between organs. Also, trauma and hemorrhage elevated water content in a majority of organs (lungs, brain, skin, liver intestines, skeletal muscle, with exceptions (kidney, stomach and testis). Furthermore, water content was higher in the traumatized tissues as compared to non-traumatized tissues (liver, intestine and skeletal muscle). Bradykinin receptor antagonism attenuated the increase in water content of the brain, but had less effect on the other organs. Blockade of the bradykinin receptor had no effect on the response of MAP and HR to trauma and hemorrhage.

**Conclusion:** Trauma and hemorrhage leads to edema (elevation in water content) in most organs, and trauma to the organ itself leads to an even greater increase in edema. Blockade of bradykinin attenuates edema in brain, suggesting that brain edema after trauma may be mediated by bradykinin.

**References**

1. Sawamura A, et al. Thromb Res. 2009;124(5):608–13.
2. Chapman MP, et al., J Trauma Acute Care Surg. 2016;80(1):16–23; discussion-5.
3. Wu X, et al. Am J Physiol Regul Integr Comp Physiol. 2016;310(4):R323–9.
4. Darlington DN, et al. Shock. 2013;39(5):440–6.
5. Marcos-Contreras OA, et al. Blood. 2016:128(20):2423–2434
6. Cap AP. Blood 2016: 128(20): 2376

## P2 Microcirculatory oxygen levels and blood flow following trauma and hemorrhage on rats

### Christopher R. Bologna^1^, Johan A. van Nispen^2^, Kim E. Hildreth^3^, David Barraza^3^, Charnae E. Williams^3^, Michael A. Dubick^3^, Ivo P. Torres Filho^3^

#### ^1^Louisiana State University, Baton Rouge, LA, 70803, USA; ^2^Washington University in Saint Louis, St. Louis, MO, 63130, USA; ^3^US Army Institute of Surgical Research, JBSA Fort Sam Houston, TX 78234, USA

##### **Correspondence: **Ivo P. Torres Filho (ivo.p.torresfilho.civ@mail.mil)

*Journal of Translational Medicine *2018, **16(Suppl 3):**P2

**Background:** Following traumatic hemorrhagic shock (HS), lack of oxygen perfusion can cause death [1]. Hemorrhagic shock causes a broad-spectrum response that changes blood flow, blood chemistry, coagulation, and oxygen perfusion [2]. Microcirculatory oxygen partial pressure (PO_2_) and blood flow measurements on a live animal through a muscle such as the cremaster, provides accurate measurements of oxygen perfusion [3]. The objective of this study was to create a rat model to determine changes in blood chemistry, coagulation, PO_2_ and microcirculatory flow following significant hemorrhage. This study uses a model that can be used in future experiments to study the addition of different adjuncts to test their effectiveness in improving microcirculatory PO_2_ and flow, thus increasing survivability.

**Materials and methods:** Rats were anesthetized with isoflurane, and the left cremaster, the thin muscle surrounding the testicle, was surgically prepared. A laparotomy and subsequent hemorrhage of 40% estimated total blood volume was performed to produce significant traumatic HS. Five blood samples were taken: time points 0 before hemorrhage (baseline), 60, 120, 180, and 210 min. Immediately before each blood sample, a PO_2_ sensor (OxyLED, Oxygen Enterprises) and a microcirculatory flow sensor (FLPI-2, Moor Instruments) provided cremaster readings. These readings were repeated 3 times at each time point. Biochemical, hematological, and coagulation tests were completed on each blood sample. Averages and frequency histograms of the microcirculatory PO_2_ and blood flow values from five experiments were analyzed and compared to other systemic data collected at each time point.

**Results:** Average PO_2_ and blood flow levels were statistically lower at all time points following hemorrhage compared to baseline. However, flow levels were statistically greater at time points 120, 180, and 210 min in comparison to the post-hemorrhage time point at 60 min, showing the animal’s response to increase flow following HS. There was an inverse relationship between microcirculatory PO_2_ and systemic lactate, with an R^2^ of 0.4114. Using Spearman’s Rank Correlation Coefficient, high and moderate correlation was found between PO_2_ and hematocrit, systemic lactate, and blood urea nitrogen, and no correlation was found between PO_2_, fibrinogen or glucose.

**Conclusions:** This model shows how microcirculatory PO_2_ and blood flow changes over time with HS. It is reproducible and applicable for future studies using compounds that may improve microcirculatory PO_2_ and flow following HS. This model is unique because it measures microcirculatory PO_2_ and flow, and helps understanding oxygen perfusion in skeletal muscle during HS.

**References**

1. McPherson R, Pincus M. Henry’s Clinical Diagnosis and Management 23rd Edition, *Laboratory Methods*, 2017; 94.
2. Torres Filho I, Torres L, Salago C, Dubick M. Plasma syndecan-1 and heparan sulfate correlate with microvascular glycocalyx degradation in hemorrhaged rats after different resuscitation fluids. Am J Physiol Heart Circ Physiol, 2016; 310: H1468–H1478.
3. Johnson PC, Vandegriff K, Tsai AG, Intaglietta M. Effect of acute hypoxia on microcirculatory and tissue oxygen levels in rat cremaster muscle. J Appl Physiol (1985), 2005; 98(4): 1177–1184.

## P3 Evaluation of Analgesic Properties of Morphine and Meloxicam

### Brittny L. Garcia^1^, Megan E. Manos^1^, Misty M. Strain^2^, Roger Chavez^2^, David Trapolsi^2^, Alex Trevino^2^, Thomas Garza^2^, Stephen Crimmins^2^

#### ^1^University of Texas at Austin, Austin, TX 78705, USA; ^2^US Army Institute of Surgical Research, JBSA Fort Sam Houston, TX 78234, USA

##### **Correspondence: **Stephen Crimmins (stephen.l.crimmins.mil@mail.mil)

*Journal of Translational Medicine *2018, **16(Suppl 3):**P3

**Background:** Burn injuries account for a large number of combat casualties, and opioids are used as one of the primary sources of medical treatment [1]. Systemic opioid use, however, can have adverse effects such as addiction, respiratory and cognitive depression, nausea, and constipation [2]. Due to adverse effects of opioids, there has been a push to reduce morphine dosages while maintaining analgesic efficacy. One class of drugs that may help extend the analgesic duration of opiates are Cox-2 inhibitors. Opiates like morphine may work synergistically with Meloxicam, a nonsteroidal anti-inflammatory drug, to produce longer lasting pain relief. [3] The objective of this study is to determine whether morphine works synergistically with Meloxicam to reduce pain after a full thickness burn.

**Materials and methods:** A rat model was used to determine the analgesic properties of meloxicam and morphine. Thermal hyperalgesia was examined before and after burn injury using the Hargreaves test. Thermal injury was induced under anesthesia, 4% isoflurane in 100% oxygen, by placing a 100 °C probe on the plantar surface of the right hind paw for 30 s. Silver sulfadiazine (1%) ointment was applied after burn injury to prevent infection. Animals were allowed to recover for 7 days post-burn to allow the pain response to peak. Prior to thermal hyperalgesia testing, animals were acclimated to the behavioral room and behavioral testing apparatus. In Experiment 1, thermal hyperalgesia was examined on Day 7 post-burn at 15, 30, and 60 min after injection of either morphine or vehicle. In Experiment 2, thermal hyperalgesia was examined at 1, 2, 3, 6 and 24 h after injection of morphine, vehicle, meloxicam, or a combination of morphine and meloxicam on Day 7. Animals were euthanized by decapitation after their last time point. Spinal cord, brain, liver, and blood samples were taken.

**Results:** In Experiment 1, morphine reduced pain sensitivity in both the injured and uninjured paw at 15 and 30 min after injection, and at 1 h in the injured paw. In Experiment 2, pre-planned comparison of morphine and vehicle showed significant analgesia at 1 h. The addition of meloxicam did not significantly extend or enhance the effect of morphine at the examined time points.

**Conclusions:** Morphine produces a significant analgesic effect, but it begins to decline after an hour of administration. This study showed no significant synergistic analgesic effect from meloxicam and morphine.

**References**

1. Fowler Marcie, Clifford L. John, Garza H. Thomas, Slater M. Terry, Arizpe M. Helen, Novak Joseph, Petz N. Lawrence, Loyd R. Dayna. A rat model of full thickness thermal injury characterized by thermal hyperalgesia, mechanical allodynia, pronociceptive peptide release and tramadol analgesia. Burns. 2014;40:579–771.
2. Küchler Sarah, Wolf B. Nadine, Heilmann Sarah, Weindl Günther, Helfmann Jürgen, Yahya Mohd Momin, Stein Christoph, Schäfer-Korting Monika. 3-D Wound healing model: influence of morphine and solid lipid nanoparticles. Biotechnology. 2010;148:24–30.
3. Abass Marwa, Mosbah Esam, Karrouf Gamal, Zaghloul Adel. Synergistic efficacy of tramadol and meloxicam on alleviation of pain and selected immunological variables after sciatic nerve ligation in rats. IJVSM. 2014;2:14–20.

## P4 The effects of IV fluid resuscitation on the gut microbiome, absorption, and inflammation post burn in swine

### Charlotte J. Winkler^1,2^, Belinda I. Gómez^1^, Matthew K. McIntyre^1^, Tony Chao^1^, Joshua S. Little^1^, Michael A. Dubick^1^, David M. Burmeister^1^

#### ^1^Damage Control Resuscitation, US Army Institute of Surgical Research, JBSA Fort Sam Houstan, TX 78234, USA; ^2^Yale University, New Haven, CT 06520, USA

##### **Correspondence: **David M. Burmeister (david.m.burmeister3.civ@mail.mil)

*Journal of Translational Medicine *2018, **16(Suppl 3):**P4

**Background:** Extensive burn injury (> 30% total body surface area [TBSA]) elicits a systemic inflammatory response that leads to multiple organ dysfunction which includes impaired intestinal function [1]. Intravenous fluid resuscitation is a cornerstone of modern burn care [2] and studies on the effect of different volumes of IV fluid on the intestinal microbiota and absorption following burn are limited.

**Materials and methods:** Anesthetized Yorkshire swine subjected to 40% TBSA thermal burns (100 °C brass probes for 30 s [3]) were randomized to one of three groups: no IV fluids (None; n = 5), or lactated Ringer’s solution at 15 ml/kg/day (low; n = 6), or 2 mlx %TBSA/kg/day (High; n = 6). On days 0, 1, and 2 fecal swabs were collected for DNA isolation and 16 s high throughput sequencing was used to characterize bacterial populations. Ileum tissue was collected after euthanasia (day 2) for Western blot, histopathology, and cytokine analyses.

**Results:** Histopathology was used to quantify the total area of goblet cells and villus height. Greater total goblet cell area was found in animals given high IV fluids (p = 0.014), whereas no difference in villus height was detected.

Although there was no significant difference in edema between groups, expression of sodium-glucose transporters (SGLT1) and aquaporin-1 (AQP1) involved in water reabsorption was greatest in animals not receiving IV fluids (p ≤ 0.05; None v. High). Caspase expression was greatest in animals not receiving IV fluids, possibly due to the larger workload placed on these cells to maintain vascular hemodynamics (p ≤ 0.05). Hsp70, an inducible stress-response was greatest in animals receiving large amounts of fluids (p = 0.05). Greater amounts of IV fluids generally correlated to lower concentrations of pro-inflammatory cytokines (Il-1α, IL-12, IL-1β, IL-6, IL-18).

Thermal injury greatly influenced microbiome alpha and beta diversity in the gut. (p = 0.023). Alpha diversity, as measured by observed operational taxonomic units and Shannon diversity, decreased regardless of treatment (p ≤ 0.05; Day 0 and 2 for none and low). Beta diversity is also altered post burn (p = 0.023). Largely, diversity did not recover to pre-burn levels. Lactobacillaces, beneficial bacteria, are reduced post burn and not affected by IV fluid. The proportion of proteobacteria, associated with inflammation of the gut, increased post burn.

**Conclusions:** Severe burns lead to changes in the gut, some of which can be influenced by amount of IV fluid resuscitation given. Higher amounts of IV fluids protect the ileum from increased cell death and inflammation but not from a decrease in microbiome diversity.

**References**

1. CPT Thmas LeVoyer, MAJ Cioffi WG, CPT Pratt L, MAJ Shippee R, COL McManus WF, Mason AD, COL Pruitt BA. Alterations in intestinal permeability after thermal injury. Arch Surg. 1992; 127(1): 26–30.
2. Guilabert P, Usua G, Martín N, Abarca L, Barret JP, Colomina MJ. Fluid resuscitation management in patients with burns: update. Br. J. Anaesth. 2016; 117(3):284–296.
3. Burmeister D, McItyre M. Baker B, Rizzo JA, Brown A, Natesan S, Chung KK, Christy RJ. Impact of isolated burns on major organs: a large animal model characterized. Shock. 2016; 46:137–147

## P5 Severe burns increase mitochondrial ROS production in human adipose-derived stem cells

### Grace Chu-Yuan Chu^1,2^, Tony Chao^1^, Belinda I. Gómez^1^, Joshua S. Little^1^, Tiffany C. Heard^1^, Michael A. Dubick^1^, Robert J. Christy^1^, David M. Burmeister^1^

#### ^1^US Army Institute of Surgical Research, JBSA Fort Sam Houston, TX 78234, USA; ^2^University of Michigan, Ann Arbor, MI 48109, USA

##### **Correspondence: **David M. Burmeister (david.m.burmeister3.civ@mail.mil)

*Journal of Translational Medicine *2018, **16(Suppl 3):**P5

**Background:** Severely burned patients (over 30% of the total body surface area) undergo a state of prolonged hypermetabolism that impairs wound healing [1]. Current research has suggested that adipose-derived stem cells (ASCs) are an attractive solution to treating burn wounds in human patients [2]. The large amount of stem cells required for treatment may be obtained from the subcutaneous adipose tissue of severely burned patients during surgical debridement [3]. While previous studies showed that severe burns alter the metabolic activity of subcutaneous adipose tissue [4], the effect of severe burns on ASCs is unknown. The purpose of this study is to determine the bioenergetic capacity of ASCs from burn patients by analyzing mitochondrial metabolic activity, mitochondrial abundance, and ROS production.

**Materials and methods:** Frozen ASCs from severely burned patients (BP, n = 6) and abdominoplasty patients (HAP, n = 6) were provided from the lab of Robert Christy. Upon reaching 80% confluency, cells were trypsinized and harvested. Flow cytometry was used to determine ASC CD90, CD105, CD73, and CD44 positive cells, mitochondrial abundance with MitoTracker Green, and ROS production with MitoSOX Red. JC-10 Mitochondrial Membrane Potential Assays were used to determine mitochondrial membrane integrity. Cell Mito Stress Tests and Glycolytic Rate Assays were performed at passage 2 using a Seahorse XFe24 Analyzer (Agilent, Santa Clara, CA). Cells were seeded in cell culture microplates and incubated in 37 °C overnight prior to the assays.

**Results:** No significant differences were found in ASC population, mitochondrial abundance, or mitochondrial membrane potential. BP ASCs had significantly higher mitochondrial ROS production (10.8 ± 1.58 vs 5.97 ± 0.368, p < 0.05) than HAP ASCs. No significant differences were observed in the mitochondrial respiration or glycolytic rates among the HAP and BP ASCs.

**Conclusions:** The higher levels of mitochondrial ROS production of ASCs from severely burned patients may suggest increased likelihood of ROS-induced oxidative damage after burn injury. This study suggests that ASCs derived from tissues of burned patients do not exhibit altered metabolic capacity. Further investigations are required to determine their use for stem cell therapy and implications in burn pathophysiology.

**References**

1. Rowan M.P., et al. Burn wound healing and treatment: review and advancements. Crit Care. 2015;19:243.
2. Cheng J. Z., et al. Therapeutic Use of Stem Cells in Treatment of Burn Injuries. J Burn Care Res. 2018; 39:175–182.
3. Chan, R. K., et al. Development of a vascularized skin construct using adipose-derived stem cells from debrided burned skin. Stem Cells Int. 2012; 2012:1–11.
4. Patsouris D., et al. Burn induces browning of the subcutaneous white adipose tissue in mice and humans. Cell Reports. 2015; 13:1538–1544.

## P6 Measurements of platelet function in vivo using a rat model of prolonged field care

### Johan A. van Nispen^1^, Christopher R. Bologna^2^, David Barraza^3^, Kim E. Hildreth^3^, Charnae E. Williams^3^, Michael A. Dubick^3^, Ivo P. Torres Filho^3^

#### ^1^Washington University in Saint Louis, Saint Louis, MO, USA; ^2^Louisiana State University, Baton Rouge, LA, USA; ^3^US Army Institute of Surgical Research, JBSA Fort Sam Houston, TX, USA

##### **Correspondence: **Ivo P. Torres Filho (ivo.p.torresfilho.civ@mail.mil)

*Journal of Translational Medicine *2018, **16(Suppl 3):**P6

**Background:** Despite advancements in care, soldiers continue to experience long extrication times, during which coagulopathy and decreased perfusion occur (1). Consequentially, developing methods to increase survival during times of prolonged field care (PFC) is of the utmost importance. To test the capability of various drugs to improve these variables, a model was developed wherein blood flow and thrombus formation could be quantified during prolonged hemorrhagic shock.

**Materials and methods:** Rats anesthetized with isoflurane underwent surgery to exteriorize the cremaster muscle. A baseline blood sample was obtained. Then the rats underwent a laparotomy to simulate trauma. The completion of trauma established the beginning of the experiment at time equal to 0 min. At 30 min post-trauma, rats were hemorrhaged (40% of total calculated blood volume) over 30 min. A post-hemorrhage blood sample was collected. The rats were then subjected to 2 mL blood draws each hour for 5 h after the initial blood sample was collected, with the blood being replaced with normal saline. 90 min into the procedure, cold stored (5 days) platelets from donor rats were fluorescently labeled and infused (approximately 10% of endogenous platelet number). At 120 min, a nitrogen laser was used to induce thrombus formation in selected venules of 17–30 µm in diameter, as described previously (2). Using confocal intravital microscopy, thrombus height and area as well as fluorescent platelet adhesion were measured off-line from video recordings. At 240 min, new recordings were made, whenever possible, to measure the same parameters off-line. The rat was euthanized humanely at the 300 min post-trauma. Rotational thromboelastometry was performed using FIBTEM and EXTEM.

**Results:** Data are reported as mean ± standard deviation. The average height of the thrombus was 11.3 ± 6.0 µm, and the average area was 265 ± 248 µm^2^, during hemorrhagic shock. The EXTEM clotting time (CT) at baseline was 41.00 ± 8.86 s, the alpha angle was 81.5° ± 1.03°, and the clot formation time was 41.13 ± 5.03 s. FIBTEM CT was 35.56 ± 7.41 s and the maximum clot firmness was 14.06 ± 2.86 mm.

**Conclusions:** A rat model can simulate the scenario of an injured soldier during delayed evacuations. Using these data, measurements of systemic coagulation function and platelet function in vivo during times of PFC are being developed which can be used in experiments to determine the effectiveness of treatments to extend survival.

**References**

1. Jacob, M, Kumar, P: The challenge in management of hemorrhagic shock in trauma. *Medical Journal, Armed Forces India*, 2014; *70*(2):163–169.
2. Torres Filho IP, Torres LN, Valdez C, Salgado C, Cap AP, Dubick MA: Refrigerated platelets stored in whole blood up to 5 days adhere to thrombi formed during hemorrhagic hypotension in rats. *J Throm Haemostasis, 2017;* 15(1):163–175.

## P7 Developing semi-reusable training models for use in prolonged field care medical simulations to test clinical decision making

### Joshua A. Fahy^1, 2^, Deana A. Apple^2^, Katy Cohen^2^, Jeremy Pamplin^2^, Maria Serio-Melvin^2^, Sena R. Veazey^2^

#### ^1^University of Arkansas, Fayetteville, AR 72701, USA; ^2^US Army Institute of Surgical Research, JBSA Fort Sam Houston, TX 78234, USA

##### **Correspondence: **Sena R. Veazey (sena.r.veazey.ctr@mail.mil)

*Journal of Translational Medicine *2018, **16(Suppl 3):**P7

**Background:** High-fidelity simulation (HFS) is the reproduction of medical scenarios through the use of a computerized manikin that is programmed to recreate clinical conditions and react to the caregivers’ actions [1]. Using high fidelity manikins as procedural task trainers is limited by cost and realism. Using models repeatedly to reduce costs reduces realism and impacts clinical decision making within the scenario because used manikin skins show indications of previous interventions. The objective of this study was to develop cost effective, realistic manikin skins that could be used to test caregiver decision and performance related to procedures commonly encountered during combat casualty care.

**Materials and methods:** Dragon Skin^®^ (Smooth-On, Macungie, PA, USA) products are mixed to form a silicone elastomer. The elasticity, color, and texture are varied to suit specific tissue types (skin, muscle, adipose, etc.) We made cricothyrotomy replacement neck skins using single layer sheets cut to size and needle decompression (NDC)/chest thoracostomy (CT) skins using two layered-sheets made to wrap around a plastic rib-cage. For the NDC/CT model, we placed balloons and sponges inside the skeleton. Procedural success penetrated the balloons, releasing air, and expanding the sponge. We assessed the realism of our skins using a paper survey that compared the tactile properties of our models and the standard manikin skin to our subjects’ recollection of human tissue during past procedures using a Likert Survey with 1–5 rating scales where 1 = not alike and 5 = alike.

**Results:** Our models were reviewed by six clinicians (critical care intensivists, nurses, and medics). The models were deemed more realistic than the standard manikin skin (2.50 ± 0.22 vs. 3.83 ± 0.16, p < 0.0001). Our cricothyrotomy skins were less expensive than the commercial models: Laerdal $47.33/skin/use [2], Syndaver $62.40/skin/use [3], ours $3.00/skin/use. Our NDC/CT skin was also less expensive than commercial models. Cost for Laerdal’s trainer is $1260.00 with reusable parts ranging from $20.30 to $106.00/procedure [4] whereas ours is estimated $85.00 ($15.00 for the skin, $5.00 for reusable materials like tape, and a one-time $65.00 cost for the skeleton rib-cage). Our models reduced costs/procedure by > 90%.

**Conclusions:** Our Dragon Skin^®^ models provide superior clinical fidelity at lower cost than similar commercial products. These advantages facilitate increased realism for procedural simulation training or assessment at a price point that allows increased frequency task performance.

**References**

1. Cortegiani A, Russotto V, Montalto F, Iozzo P, Palmeri C, Raineri SM, et al. (2015) Effect of high-fidelity simulation on medical students’ knowledge about advanced life support: a randomized study. PLoS ONE 10(5): e0125685. 10.1371/journal.pone.0125685
2. “Neck Skin Kit (6).” Trachlight™ End of Life Announcement, Laerdal Medical, http://www.laerdal.com/us/item/212-21050.
3. “Adult Cric Trainer.” SynDaver Labs, syndaver.com/shop/synatomy/deluxe-cric-trainer/
4. “Pneumothorax Trainer.” Trachlight™ End of Life Announcement, Laerdal Medical, http://www.laerdal.com/us/doc/983/Pneumothorax-Trainer.

## P8 Descriptive analysis of military canine trauma

### Kerry E. Gonyeau^1^, Emily A. Howard^2^, Thomas H. Edwards^3^, Laura L. F. Scott,^3^, Jacque Parker^4^, Kelly Hall^5^

#### ^1^Texas Tech University, Lubbock, TX, 79409, USA; ^2^Texas A&M University College of Veterinary Medicine, College Station, TX, 77843, USA; ^3^US Army Institute of Surgical Research, JBSA Fort Sam Houston, TX 78234, USA; ^4^Holland Memorial Military Working Dog Hospital, JBSA-Lackland AFB, TX, 78236, USA; ^5^University of Minnesota College of Veterinary Medicine, St. Paul, MN, 55108, USA

##### **Correspondence: **Thomas H. Edwards (thomas.h.edwards.mil@mail.mil)

*Journal of Translational Medicine *2018, **16(Suppl 3):**P8

**Background:** Military Working Dogs (MWDs) have been used in many theaters of operations to improve the security of our servicemen and women [1]. These canines incur a wide range of traumatic injuries, sometimes leading to death [2]. One study investigated gun shot wound (GSW) injuries; however, no single study has attempted to describe all forms of trauma [3]. Because most injuries obtained by human military personnel are penetrating, we hypothesized the same to be true for MWDs. Our objective was to identify traumas obtained by MWDs throughout CENTCOM since September 11, 2001. The results of this study will help to detect capability gaps and optimize management of traumatic injuries for canines while in theater.

**Materials and methods**: MWD traumas occurring in the CENTCOM AOR from September 11, 2001 to June 1, 2018 were identified from the following sources: an unpublished masters thesis, aeromedical evacuation logs, admission logs at Dog Center Europe, personal records from military veterinary clinical specialists, and Special Operations Forces veterinarians. Retrospective information from medical records, death certificates, necropsy reports, and blood work was entered into Research Electronic Data Capture (REDCap), an online database. Summary statistics were estimated from relevant demographic and injury characteristics. Differences between groups were assessed using the Wilcoxon rank sum test, Chi square test, or the *z* score test for two population proportions using an alpha of 0.05.

**Results:** We found 135 cases of traumas spanning 109 MWDs. The majority (107) had either solely penetrating (93) or a combination of penetrating and blunt/burn (14) injuries. A total of 21 MWDs died in theater; 16 were killed in Afghanistan, 3 in Iraq, 1 in Djibouti, and 1 was listed only as CENTCOM. Multiple traumas occurred in 21 dogs; 44% of these injuries were from knives/sharp objects and the majority of these dogs (18) had explosive detection certifications. Overall, leading causes of injuries were lacerations from knives/sharp objects (n = 47), bites/scratches (n = 30), and explosions (n = 26).

**Conclusions:** This study provides the first evidence describing a collection of all forms of trauma sustained by MWDs in theater. Penetrating wounds were found to be the main cause of injury. In consideration of the number of traumas, few deployed dogs died from their injuries with most of these casualties occurring in Afghanistan. These results are helpful in educating veterinarians and human healthcare providers about the nature of wounds that MWDs encounter in combat operations, allowing for more efficient treatment in the future.

**References**

1. Orman, J. A., Parker, J. S., Stockinger, Z. T., & Nemelka, K. W. (2018). The Need for a Combat Casualty Care Research Program and Trauma Registry for Military Working Dogs. *Military Medicine*.
2. Miller, L., Pacheco, G. J., Janak, J. C., Grimm, R. C., Dierschke, N. A., Baker, J., & Orman, J. A. (2018). Causes of Death in Military Working Dogs During Operation Iraqi Freedom and Operation Enduring Freedom, 2001–2013. *Military Medicine*.
3. Baker, J. L., Havas, K. A., Miller, L. A., Lacy, W. A., & Schlanser, J. (2013). Gunshot wounds in military working dogs in Operation Enduring Freedom and Operation Iraqi Freedom: 29 cases (2003–2009). *Journal of Veterinary Emergency and Critical Care, 23*, 47–52.

## P9 Hypoxic (3% O_2_) preconditioning does not alter in vitro function of human mesenchymal stem cells

### Laurynn Garcia^1^, Christopher Delavan^2^, Carolina Cantu^2^, James A. Bynum^2^, Andrew P. Cap^2^, Maryanne C. Herzig^2^, Barbara Christy^2^

#### ^1^Duke University, Durham, NC 27708, USA; ^2^Coagulation and Blood Research, US Army Institute of Surgical Research, JBSA-Fort Sam Houston, TX 78234, USA

##### **Correspondence: **Barbara Christy (barbara.christy3.ctr@mail.mil)

*Journal of Translational Medicine *2018, **16(Suppl 3):**P9

**Background:** Mesenchymal stem cells (MSCs) show promise as cell therapy agents for treatment of traumatic injury [1]. MSCs demonstrate anti-inflammatory and immunomodulatory activities thought to be mediated through a paracrine mechanism [2]. However, safety considerations need further exploration. MSCs exhibit pro-coagulant activity roughly correlating with tissue factor (TF) expression [3], which may create additional stress in coagulopathic patients. MSC culture is generally performed under normoxic conditions, while most MSCs reside in vivo in hypoxic niches [4]. Studies suggest hypoxic preconditioning of MSCs may promote survival and efficacy in vivo [5,6]. This study investigates the effects of preconditioning human bone marrow (BM-MSCs) and adipose MSCs (AD-MSCs) with hypoxia (3% O_2_) or normoxia (21% O_2_) for 24 h on in vitro measures of safety and potency.

**Materials and methods:** MSC populations were pre-conditioned by incubation for 24 h in normoxic (21% O_2_) or hypoxic (3% O_2_) conditions; all assays were performed under normoxic conditions. Pro-coagulant activity of MSCs in platelet poor human plasma was measured by thromboelastography, and cell surface TF was determined by flow cytometry. Immunomodulation activity was measured using a mixed lymphocyte reaction assay by addition of PBMCs to pre-conditioned MSCs and incubation with or without PHA stimulation for 72 h. Relative PBMC and MSC numbers were determined using a luminescent ATP assay. IDO enzyme activity in response to inflammatory signaling was evaluated by measuring the product kynurenine secreted in 24 h.

**Results:** Both normoxia- and hypoxia- exposed MSCs reduced time to clot initiation. No significant difference in coagulation time for MSCs preconditioned in hypoxia vs. normoxia was observed. The percentage of AD-MSCs expressing TF was unchanged after hypoxia, while BM-MSCs showed a 10% decrease in cells expressing surface TF. The MLR showed no significant difference in PBMC suppression following MSC preconditioning in hypoxia or normoxia, with both demonstrating higher PBMC suppression as MSC number increased. IDO activity was not significantly different in MSCs pre-incubated in hypoxia vs. normoxia, with both responding similarly to inflammatory cytokine stimulation (see Table 1).

**Table:** Table 1

	Ad-MSCs		BM-MSCs	
	Normoxia	Hypoxia	Normoxia	Hypoxia
TEG-R time (min)	3.3 ± 0.28	3.0 ± 0.14	3.65 ± 0.07	3.9 ± 0.14
Flow cytometry-TF % expression	93.7%	96.4%	38.2%	28.3%
MLR-% PBMC Suppression (2.5 PBMC: 1 MSC)	97.23% ± 2.96	99.76% ± 2.38	123.63% ± 2.91	116.10% ± 9.70
IDO-Kynurenine Production (µg/ml) (w/TNFα + IFNγ)	5.45 ± 0.02	5.28 ± 0.01	5.11 ± 0.08	5.34 ± 0.15

**Conclusions:** No significant effect was seen with hypoxic preconditioning on human MSCs in terms of pro-coagulant activity, PBMC suppression, or IDO activity under these conditions. While TF levels were not affected in AD-MSCs, BM-MSCs exposed to hypoxia showed decreased TF levels. This observation could have important safety implications. Future study is required to confirm this finding. It is possible that lower O_2_ levels or longer treatment times may show more effect and requires additional study.

**References**

1. Matthay M, Pati S, Lee J. Concise Review: Mesenchymal Stem (Stromal) Cells: Biology and Preclinical Evidence for Therapeutic Potential for Organ Dysfunction Following Trauma or Sepsis. Stem Cells. 2017; 35: 316–324.
2. Gebler A, Zabel O, Seliger B. The immunomodulatory capacity of mesenchymal stem cells. Trends Mol Med. 2012; 18: 128–134.
3. Christy B, Herzig M, Montgomery R, Delavan C, Bynum J, Reddoch K, Cap A. Pro-coagulant activity of human mesenchymal stem cells. J Trauma Acute Care Surg. 2017; 81: S164–169.
4. Ahmed M, Garzón-Muvdi T, Quiñones-Hinojosa A. Oxygen in Stem Cell Biology: A Critical Component of the Stem Cell Niche. Cell Stem Cell 2010; 7:150–161.
5. Beegle J, Lakatos K, Kalomoiris S, Stewart H, Isseroff R, Nolta J, Fierro F. Hypoxic Preconditioning of Mesenchymal Stromal Cells Induces Metabolic Changes, Enhances Survival, and Promotes Cell Retention In Vivo. Stem Cells. 2012; 33: 1818–1828.
6. Chacko S, Ahmed S, Selvendiran K, Kuppusamy M, Khan M, Kuppusamy P. Hypoxic preconditioning induces the expression of prosurvival and proangiogenic markers in mesenchymal stem cells. Am J Physiol Cell Physiol. 2010; 299: C1562–C1570.

## P10 Roles of A_2B_ and A_3_ adenosine receptors in adenosine-induced anti-platelet aggregation

### Luke A. del Balzo^1^, Maria E. Ramos^1^, Bunyen Teng^2^, Daniel N. Darlington^2^, Andrew P. Cap^2^

#### ^1^Emory University, Atlanta, GA 30322, USA; ^2^US Army Institute of Surgical Research, JBSA Fort Sam Houston, TX 78234, USA

##### **Correspondence: **Daniel N. Darlington (daniel.n.darlington.civ@mail.mil)

*Journal of Translational Medicine *2018, **16(Suppl 3):**P10

**Background:** Adenosine, an autacoid and metabolite of adenosine triphosphate (ATP), has been known to induce anti-platelet aggregation. Four adenosine G protein-coupled surface receptors (ARs), A_1_, A_2A_, A_2B_ and A_3_, are implicated with the release of adenosine that follows a traumatic injury [1]. Previous studies from our group and others demonstrated that the A_2A_ AR is the predominant AR in the anti-platelet effect of adenosine [2]. The A_2B_ AR has been shown to mediate adenosine-induced anti-platelet function, but its effect has not been shown in human [3]. The antiplatelet effects of the A_1_ and A_3_ AR have not been clearly defined, especially in humans. Earlier studies have demonstrated that adenosine activates adenylyl cyclase to increase intraplatelet cAMP concentration and subsequently inhibit platelet aggregation.

**Objective:** To investigate the effect of adenosine receptor activation on ADP-induced platelet aggregation

**Methods**: Whole blood was collected over citrate from normal human volunteers (Protocol L-013-009) and platelet rich plasma (PRP) was generated by gentle centrifugation (200×*g* for 10 min, no brakes) and extracted. Platelet poor plasma (PPP) was generated by centrifugation of the remaining blood (3000×*g* for 10 min). Platelets were diluted in PPP to a platelet count of 100,000/μl. Light transmission aggregometry (Synergy Neo2, BioTek) was performed by 1 mM ADP with or without NECA (non-specific AR agonist), CGS 21680 (A_2A_ AR agonist), BAY 60-6583 (A_2B_ AR agonist), DPCPX (A_1_ AR antagonist), Sch 58261 (A_2A_ AR antagonist), GS 6201 (A_2B_ AR antagonist), and MRS 1220 (A_3_ AR antagonist). Cyclic adenosine monophosphate (cAMP) was measured via liquid chromatography-tandem mass spectrometry (Quantiva, ThermoFisher).

**Results**: The adenosine agonist, NECA, inhibited ADP-induced platelet aggregation. Blockade of both A_2A_ and A_3_ reversed the effects of NECA. Blockade of A_2B_ had no effect. Blocking the A_1_ AR enhanced the effects of NECA. NECA elevated cAMP in platelets, and blockade of A_2A_ inhibited this increase. Blockade of A_1_ had no effect on the NECA-induced rise in intraplatelet cAMP concentration.

**Conclusion:** Adenosine inhibits platelet aggregation by stimulating the A_2A_ and A_3_ AR. This inhibition is likely due to the elevation in cAMP through A_2A_. The A_2B_ AR does not play a significant role in the anti-platelet effect of adenosine in normal human platelets. The A_1_ and A_3_ AR may have a modulating effect in adenosine-induced anti-platelet function.

**References**

1. Haslam, R.J. and G.M. Rosson, Mol Pharmacol, 1975. 11(5): 528-44.
2. Fuentes, E., et al., PLoS One, 2014. 9(11): e112741.
3. Yang, D., et al., J Thromb Haemost, 2010. 8(4): 817–27.

## P11 Effects of 2-deoxy-d-glucose on platelet aggregation

### Maria E. Ramos^1^, Luke A. Del Balzo^1^, Jeffrey D. Keesee^2^, Josue Garciamarcano^2^, Xiaowu Wu^2^, Daniel N. Darlington^2^, Andrew P. Cap^2^

#### ^1^Emory University, Atlanta, GA 30322, USA; ^2^US Army Institute of Surgical Research, JBSA Fort Sam Houston, TX 78234, USA

##### **Correspondence: **Daniel N. Darlington (daniel.n.darlington.civ@mail.mil)

*Journal of Translational Medicine * 2018,**16(Suppl 3):**P11

**Background:** Acute coagulopathy, caused by severe trauma and hemorrhage, is characterized by a decrease in clotting firmness, platelet dysfunction and ischemia [1–4]. Platelets are highly energetic cells that require ATP for all active processes of aggregation and contribute 70–80% of clot strength [5]. Creating a strategy to alleviate the progression of coagulopathy begins with studying the intracellular mechanisms that modulate platelet aggregation. Previous studies have observed a decrease in platelet ATP and increase in platelet dysfunction after both trauma and hemorrhage in rats. It is unknown, however, if the decrease in ATP directly triggers platelet dysfunction. This study aimed to determine if a fall in intracellular ATP affects platelet aggregation to natural stimuli.

**Materials and methods:** Whole blood was collected over citrate from normal human volunteers and platelet rich plasma (PRP) generated by gentle centrifugation (200*g* for 10 min, no brakes). Platelet poor plasma (PPP) was generated by centrifugation 20 KG for 10 min. PRP was diluted with PPP to a platelet count of 300,000/ml. Platelet aggregation was measured in a 96 well plate containing 168 μl of PRP, 20 μl of agonist, 10 μl of 2-deoxy-d-glucose (2DG), and 2 μl of water. 2DG is a sugar that cannot be metabolized and lowers ATP levels. Agonists used to stimulate aggregation included ADP (1 mM), U46619 (10 mM), PAR1 (1 mM), and collagen (1 mg/ml). Aggregation was measured by an increase in turbidity, by absorbance at λ = 550 nm before, and up to, 60 min after the addition of agonist.

**Results:** 2 Deoxy-d-glucose severely attenuated or entirely inhibited platelet aggregation in response to ADP, PAR1 and U46119. We observed no effect of 2DG on platelet aggregation in response to collagen.

**Conclusions:** Human platelet aggregation in response to natural stimuli (ADP, thrombin and thromboxane) is inhibited by the introduction of 2DG. 2DG works to inhibit aggregation by decreasing the production of ATP, a crucial component of platelet aggregation. Because collagen-induced stimulation showed no response to 2DG, collagen-induced platelet aggregation may require a much smaller amount of energy (in the form of ATP) compared to other natural agonists.

**References**

1. Darlington DN, Craig T, Gonzales MD, Schwacha MG, Cap AP, Dubick MA. Shock (2013) 39, 440.
2. Wu X, Darlington DN, Cap AP. Am J Physiol Regul Integr Comp Physiol (2016) 310, R323.
3. Smolenski A. Journal of Thrombosis and Haemostasis (2012) 10,167.
4. Li, Z, Delaney MK, O’Brien KA, Du X. Arterioscler Thromb Vasc Biol 2010:30:2341.
5. Ravi, S, Chacko B, Sawada H, Kramer PA, Johnson MS, Benavides GA, O’Donnell V, Marques MB, Darley-Usmar VM. PLoS ONE 10(4): e0123597. 10.1371/journal.pone.0123597

## P12 Extraction of gut microbiota from stressed and burned rats

### Megan Manos^1^, Brittny Garcia^1^, Natasha M Sosanya^2^, Sirima Tongkhuya^2^, Misty M Strain^2^, Stephen L Crimmins^2^

#### ^1^University of Texas at Austin, Austin, TX 78705, USA; ^2^US Army Institute of Surgical Research, JBSA Fort Sam Houston, TX 78234, USA

##### **Correspondence: **Stephen L Crimmins (stephen.l.crimmins.mil@mail.mil)

*Journal of Translational Medicine *2018, **16(Suppl 3):**P12

**Background:** The human gastrointestinal (GI) tract contains over 10^14^ microorganisms that assist the body in a variety of functions including digestion and immune function [1]. These gut microorganisms can interact through the gut brain axis, a bidirectional communication system between the GI tract and the central nervous system (CNS) [1], via immune cells, neuronal pathways, or blood–brain barrier permeable neuroactive metabolites [2]. Dysregulation of gut flora can cause dysbiosis, a state where beneficial bacteria are depleted or overpowered by pathogenic bacteria [2]. Recent studies have shown that dysbiosis increases after burn injury and possibly contributes to the development of sepsis [3]. This may be due to the down regulation of epithelial cell tight junction genes, which allows for opportunistic bacteria to translocate from the gut to peripheral tissue, complicating treatment [3]. In addition, stress can increase dysbiosis and directly impact neurological diseases such as depression and anxiety as well as visceral pain severity [1,4]. The aim of this study was to examine microbiota changes after a combination of burn injury and stress. We believe the combination of stress and burn will disrupt the gut microbial profile, which will be correlated to increased pain behaviors.

**Materials and methods:** First we compared two methods of DNA extraction. Fecal pellets from pair housed rats were divided into two samples for analysis with *Qiamp PowerFecal DNA* (10, 25, 100, or 250 mg) and *EZ1 DNA Tissue* kit (10 or 25 mg). To examine the effects of stress, fecal pellets from pair housed female rats were compared between the first day of and 2 weeks after chronic unpredicted stress (i.e. forced swim, cold, sound, and restraint). Animals in the same groups (i.e., stress/no stress) were housed together. DNA was extracted using the *Qiamp PowerFecal DNA* Kit (100 mg). After DNA extraction, the concentration as well as the protein and organic material contamination were examined using spectrophotometry.

**Results:** While both kits yielded similar concentrations and amount of organic contamination, the *EZ1 DNA Tissue* Kit had more protein contamination compared to the *Qiamp PowerFecal DNA* Kit. DNA extracted from experimental sample using the *Qiamp PowerFecal DNA* Kit produced an adequate yield and purity for later PCR amplification and microbiome analysis.

**Conclusions:** Overall, the *Qiamp PowerFecal DNA* kit provided an adequate amount of DNA with less protein contaminates. Future studies, will use DNA extracted from experimental samples to examine the relationship between gut microbiota, stress, and pain.

**References**

1. Moloney, Desbonnet, Clarke, Dinan, Cryan. The microbiome: stress, health, and disease. Mamm Genome. 2014; 25:49–74.
2. Kigerl, Hall, Wang, Mo, Yu, Popovich. Gut dysbiosis impairs recovery after spinal cord injury. JEM. 2016; 213;2603–2620.
3. Earley, Akhatar, Green, Naqib, Khan, Cannon, Hammer, Morris, Li, Eberhardt, Gamelli, Kennedy, Choudhry. Burn Injury Alters the Intestinal Microbiome and Increases Gut Permeability and Bacterial Translocation. PLoS One. 2015; e0129996.
4. Rousseaux, Thuru, Gelot, Barnich, Neut, Dubuqouy, Dubuqouy, Merour, Geboes, Chamaillard, Ouwehand, Leyer, Carcano, Colombel, Ardid, Desreumaux. Lactobacillus acidophilus modulates intestinal pain and induces opioid and cannabinoid receptors. Nat. Med. 2007; 13:35–37.
5. Sosanya NM, Garza TH, Stacey W, Crimmins SL, Christy RJ, Cheppudira BP. Involvement of brain-derived neurotrophic factor in chronic intermittent stress-induced enhanced mechanical allodynia in a rat model of burn pain. (2018). Submitted.

## P13 Morphine-loaded keratin hydrogel drug release study

### Lucy J. Shaffer^1^, Christine J. Kowalczewski^2^, Robert Christy^2^

#### ^1^Oregon State University, Corvallis, OR 97333, USA; ^2^US Army Institute of Surgical Research, JBSA Fort Sam Houston, TX 78234, USA

##### **Correspondence: **Robert Christy (robert.j.christy12.civ@mail.mil)

*Journal of Translational Medicine *2018, **16(Suppl 3):**P13

**Background:** Partial thickness burns, commonly referred to as second degree burns, are painful and heal by reepithlialization. The current gold standard for pain management for burn victims is administration of intravenous opioids such as morphine. However, systemic opioid deliver causes side effects like respiratory suppression and delayed wound healing [1, 2]. However, recent finding have shown that topical morphine administration may stimulate angiogenesis and keratinocyte migration. Therefore, topical morphine administration can be achieved by loading the opioid into a biomaterial such as keratin, a strong filamentous protein found in human hair. In previous studies, keratin hydrogels have successfully shown controlled and sustained drug release to burn wounds [3]. To determine the morphine release kinetics and keratin protein breakdown characteristics of the hydrogel, a morphine dose study was performed.

**Materials and Methods:** 10 mg/ml of morphine hydrochloride was used directly or diluted to 1 or 5 mg/ml with sterile PBS to rehydrate sterile keratin powder to form a 15% weight:volume hydrogel. Unloaded (PBS only) keratin gel served as a negative control. 250 µl of each respective gel (n = 3) was added to a 1.5 ml conical tube and centrifuged briefly. Keratin hydrogels were allowed to incubate overnight. The next day, 250 µl of sterile PBS was added on top of each hydrogel. At preassigned time points (1.5, 3, 6, 12 h, and days 2–7), the PBS was removed, frozen at − 20 °C for future analysis, and replaced with fresh sterile PBS. Morphine release from the hydrogel was determined using competitive Morphine ELISA. Keratin breakdown was determined by BCA protein analysis. Repeated measures ANOVA with Tukey post hoc test measured significance across all variables over time.

**Results:** Increasing the morphine concentration loaded into the keratin hydrogel significantly increased the hydrogel degradation rate in a dose-dependent manor (Fig. 1). Morphine release from the hydrogel was sustained over the 1 week experiment and was dependent on the initial concentration and hydrogel degradation rate (Fig. 2).

**Fig. 1:**
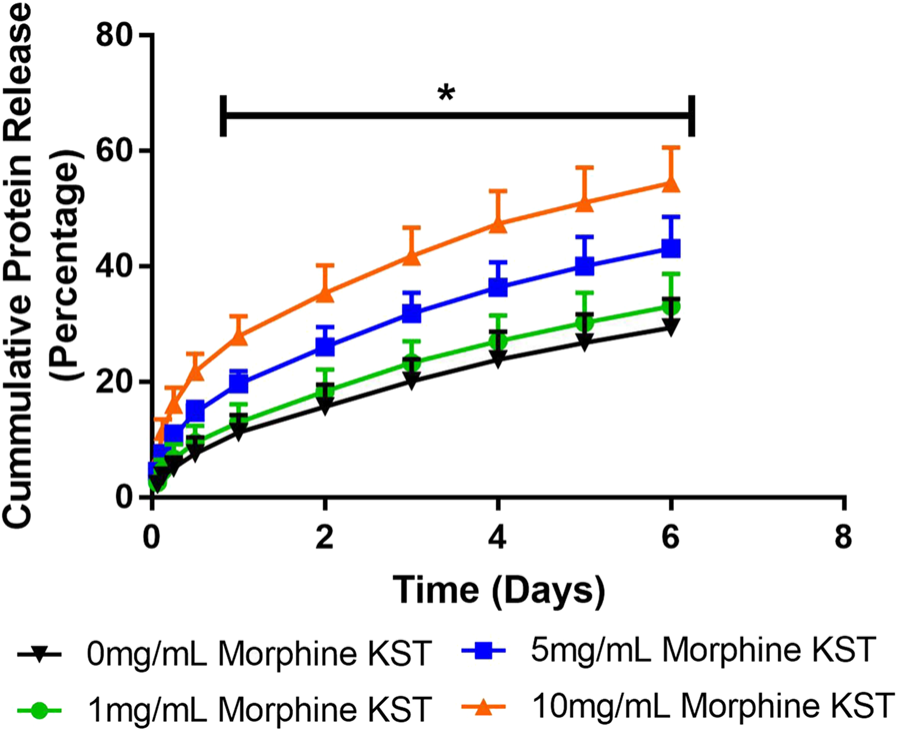
Protein degradation rate of keratin hydrogels

**Fig. 2:**
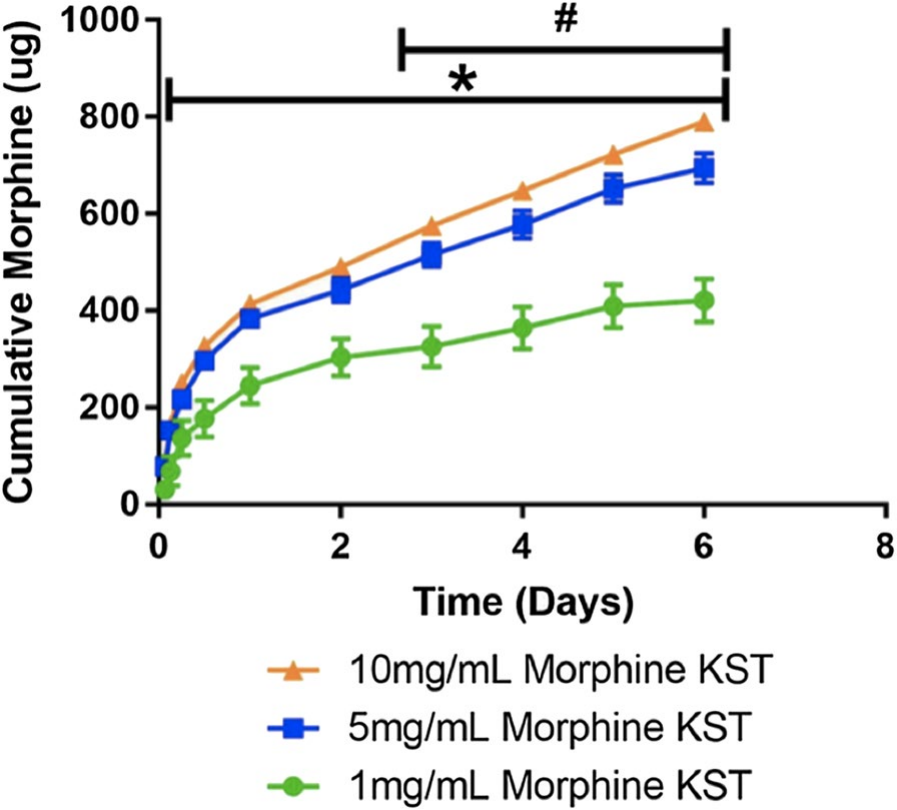
Sustained release of morphine from morphine-loaded keratin hydrogel

**Conclusions:** Various concentrations of morphine-loaded keratin hydrogel can achieve sustained and controlled delivery of morphine over a 1 week period. Topical application of a morphine-loaded keratin hydrogel has the potential to reduce pain associated with burn injuries as well as improved wound healing.

**References**

1. Breederveld RS, Tuinebreijer WE. Incidence, Cause and Treatment of Burn Casualties Under War Circumstances. Eur J Trauma Emerg Surg. 2009; 35, 240–243.
2. Küchler S, Wolf NB, Heilmann S, Weindl G, Helfmann J, Yahya MM, et al. 3D-Wound Healing Model: Influence of Morphine and Solid Lipid Nanoparticles. J Biotechnol. 2010;148(1):24–30.
3. Roy DC, Tomblyn S, Isaac KM, Kowalczewski CJ, Burmeister DM, Burnett LR, Christy RJ. Ciprofloxacin-loaded keratin hydrogels reduce infection and support healing in a porcine partial-thickness thermal burn. Wound Repair & Regeneration. 2016; 24, 657–668.

